# Electroacupuncture in the Contralesional Hemisphere Improves Neurological Function Involving GABA in Ischemia–Reperfusion Injury Rats

**DOI:** 10.1155/2021/5564494

**Published:** 2021-07-09

**Authors:** Chung-Hsiang Liu, Wen-Ling Liao, Shan-Yu Su, Wei-Liang Chen, Ching-Liang Hsieh

**Affiliations:** ^1^Department of Neurology, China Medical University Hospital, Taichung 40447, Taiwan; ^2^Graduate Institute of Integrated Medicine, College of Chinese Medicine, China Medical University, Taichung 40402, Taiwan; ^3^Center for Personalized Medicine, China Medical University Hospital, Taichung 40447, Taiwan; ^4^Department of Chinese Medicine, China Medical University Hospital, Taichung 40447, Taiwan; ^5^Division of Medical Genetics, University of Washington Medical Center, Seattle, WA 98195-7720, USA; ^6^Chinese Medicine Research Center, China Medical University, Taichung 40402, Taiwan; ^7^Graduate Institute of Acupuncture Science, College of Chinese Medicine, China Medical University, Taichung 40402, Taiwan

## Abstract

This study investigated the effect and mechanism of electroacupuncture (EA) on the contralesional hemisphere in rats with ischemic stroke. EA of 2 Hz was applied on the contralesionally Luoque (BL8) and Tongtian (BL7) acupoints of the scalp to investigate the neurological status and mechanism in ischemia–reperfusion injury rats. The differences in the neurological deficit score and Rotarod test time between days 3 and 15 after reperfusion were significantly lower in the sham group (0.00 (−1.00, 0.00) and 3.53 (−0.39, 7.48) second, respectively) than in the EA group (−4.00 (−4.00, −3.00) and 44.80 (41.69, 54.13) second, respectively, both *p* < 0.001). The ratio of infarction volume was 0.19 ± 0.04 in the sham group greater than 0.07 ± 0.04 in the EA group (*p* < 0.001). On day 15, in the cerebral cortex of the lesioned hemisphere, the gamma-aminobutyric acid (GABA)-A/actin ratio in the normal group (1.11 ± 0.36) was higher than that in the sham group (0.38 ± 0.07, *p* < 0.05) and similar to that in the EA group (0.69 ± 0.18, *p* > 0.05); the difference between the EA and sham groups was significant (*p* < 0.05). EA of 2 Hz on the BL8 and BL7 acupoints on the contralesional scalp can improve motor function and also can reduce infarction volume, and this effect of EA, and that GABA-A, plays at least a partial role in ischemia–reperfusion injury rats.

## 1. Introduction

As part of stroke epidemiology in southwestern China, an analysis of 16,892 people over 40 years of age revealed that 3.1% of them have had a stroke; of these individuals, 17.1% were found to have risk factors for stroke, such as hypertension, dyslipidemia, and diabetes [1]. Stroke is the fourth leading cause of death and the main cause of chronic disability and dementia in the United States [2, 3]. Stroke is divided into ischemic stroke and hemorrhagic stroke [4]. A clinical trial revealed that although the administration of intravenous recombinant tissue plasminogen activator (t-PA) within 3 h of ischemic stroke onset can improve clinical outcomes at 3 months, it can also increase the incidence of intracerebral hemorrhage [5]. Therefore, it is critical to develop an effective and safe method.

Cerebral edema commonly occurs in the early period after ischemic stroke and is related to the degree of neurological deficit [6]; moreover, cerebral blood flow is reduced in the penumbra of the infarct [7]. Therefore, improvement of neurological function in the early poststroke period necessitates strategies for reducing cerebral edema and preventing penumbral infarction. Cramer and Moore using functional magnetic image resonance revealed that cortical reorganization along the periphery of the infarct plays a critical role in poststroke sensorimotor functional recovery [8]. The activity of the contralesional motor cortex is vital for poststroke motor function recovery of the paretic limb [9]. The balance of interhemispheric inhibition changes after stroke. A reduction in the inhibition from the lesioned hemisphere to nonlesioned hemisphere and in the excitability of perilesional tissue is observed, resulting in a stronger inhibition from the nonlesioned hemisphere to lesioned hemisphere after stroke, which affects poststroke recovery [10]. Noninvasive cortical stimulation, including repeat transcranial magnetic stimulation and transcranial direct current stimulation, can modulate cortical excitability by, for instance, increasing the excitability of the lesioned hemisphere or decreasing the excitability of the nonlesioned hemisphere [11, 12]. In summary, the interhemispheric balance is disrupted after stroke, and the nonlesioned hemisphere shows greater inhibition of the lesioned hemisphere.

Gamma-aminobutyric acid (GABA) is an inhibitory neurotransmitter in the central nervous system. A magnetic resonance spectroscopy study revealed that, at 3–6 months after stroke, GABA levels in patients with stroke were lower than those in the healthy population [13]. GABA can modulate motor cortical plasticity and act as a biomarker for stroke recovery [14]. In the reperfusion period after cerebral ischemia, the causes of neuronal death include glutamate release of the astrocytes, Ca2+ into intracellular space, and the generation of reactive oxygen species. OX26-PEG-coated gold nanoparticles (GNPs) (OX26@GNPs) are not suitable nanocarriers for the treatment of ischemia stroke due to the fact that they can enhance the generation oxidative stress [15].

Acupuncture has been used to treat numerous diseases for at least 3000 years in China and some other Asian countries. However, its underlying mechanism remains unclear. Many studies find electroacupuncture (EA) applied to Baihui (GV20) and Dazhui (GV14) was demonstrated to reduce neurological deficit and infarct volume, increase the levels of brain-derived neurotrophic factor (BDNF), and reduce the levels of S100B in transient middle cerebral artery (MCA) occlusion rats [16, 17]. In traditional Chinese medicine, the Yellow Emperor's Classic of Internal Medicine records that left disease cures right and right disease cures left. Acupuncture can improve the flow of Qi along the meridians and balance the yin and yang [18] by regulating the balance of excitement and inhibition between the two cerebral hemispheres. Therefore, this study investigated the mechanism and effect of EA on the contralesional hemisphere in rats with ischemic stroke. In this study, the right MCA of Sprague-Dawley rats was occluded for 30 min followed by reperfusion to establish an ischemic stroke in rats.

## 2. Materials and Methods

### 2.1. Animals

Male Sprague-Dawley rats weighing 250–350 g were purchased from BioLASCO Taiwan and raised in the Animal Center of China Medical University. The animals were received care in accordance with the regulations of the Laboratory Animal Committee of China Medical University. The rats were placed in a regular 12 h light/12 h dark environment, with temperature maintained at 22–24°C and humidity maintained at 50%–70% using an air conditioner. The rats were provided with adequate feed and drinking water, and the litter was kept clean. All experimental procedures were in accordance with the regulations of the animal experiment ethics committee, and all efforts were made to try to reduce the pain or discomfort of animals.

### 2.2. Establishment of the Animal Model of Ischemic Stroke

An animal model of ischemic stroke in rats was established through intraluminal suture occlusion of the MCA, as described in our previous study [19].

First, rats were anesthetized with isoflurane gas and turned to rest in a supine position. Next, an incision was made from the midline of the neck, and the right internal carotid artery, external carotid artery, and common carotid artery were separated. Then, the pterygoid artery was ligated at its origin from the maxillary artery, the right internal carotid artery and common carotid artery were clamped in an appropriate location, and the external carotid artery was permanently ligated. A small orifice was made near the common carotid artery with a pair of small scissors. Next, a 3–0 nylon filament suture was used, and the tip of the nylon thread was heated and coated with poly-L-lysine (UNIK, Taiwan) for smooth insertion into the orifice created in the external carotid artery; the nylon thread was advanced into the internal carotid artery through the common carotid artery and moved approximately 23–25 mm. Blood flow to the MCA was thus blocked. Thereafter, the nylon thread was fixed with an arterial clip, and the nylon thread was withdrawn 30 min later, allowing reperfusion of the right MCA. After ligating the opening of the external carotid artery up and down, all arterial clamps were removed, the ligation at the pterygoid artery was removed, the skin of the rat's neck was sutured, and the rat was placed in a cage.

The rats were selected to enter the experiment only when they met the standard after learning and memory training. First, they were evaluated for neurological deficit scores at day 3 after reperfusion and then the Rotarod test followed the passive avoidance test.

### 2.3. Grouping

In this study, 24 ischemia-reperfusion injury rats with neurological deficit scores of 7–9 were randomly divided into sham and EA groups, with twelve rats in each group, and the other normal group comprised twelve normal rats without ischemia-reperfusion.

#### 2.3.1. Normal Group

The rats' neck was incised from the midline to expose the common carotid artery, and the wound was sutured 30 min later. At day 3 after operation, the rats' neurological status was evaluated, including neurological deficit score and performance on the Rotarod test and passive avoidance test. The rats were then anesthetized under isoflurane for 20 min three times a week for 2 consecutive weeks; their neurological status was evaluated again on day 15. Finally, the rats were killed, and their brains were removed: six rats for Western blot analysis and the other six rats for 2,3,5-triphenyltetrazolium chloride (TTC) stain for the measurement of cerebral infarction volume.

#### 2.3.2. Sham Group

The rats' neurological status was evaluated, including neurological deficit score and performance on the Rotarod test and passive avoidance test at day 3 after reperfusion. Next, with the rats under isoflurane anesthesia, two stainless steel acupuncture needles were inserted into subcutaneous tissue of the contralesional scalp, a location which is equivalent to the Luoque (BL8) acupoint, which serves as an anode, and the Tongtian (BL7) acupoint, which serves as a cathode in humans. The needles were connected to an EA apparatus (Trio 300, Ito, Japan) for 20 min three times a week for 2 consecutive weeks, but no electrical stimulation was delivered. The rats' neurological status was evaluated again on day 15 after reperfusion. Finally, the rats were killed, and their brains were removed: six rats for Western blot analysis and the other six rats for TTC stain.

#### 2.3.3. EA Group

The method was the same as that of the sham group, but 2 Hz electrical stimulation was delivered. The intensity of stimulation was adjusted until visible muscle contractions were seen.

### 2.4. Neurological Status Evaluation

#### 2.4.1. Neurological Deficit Score

Neurological deficit was evaluated using the modified neurological severity score [20], which was assessed by a well-trained person who was blinded to the groups. In summary, the total neurological deficit score was 18 including motor test (0–6), sensory test (0–2), beam balance test (0–6), and reflex test (0–4) (Table 1).

#### 2.4.2. Rotarod Test

The rat was placed on the shaft of a Rotamex roller treadmill (Columbus Instrument, Ohio, USA) maintained at 4 revolutions per minute (rpm) (the switch was turned on at this time). Next, the start switch was pressed, and the machine increased by 1 every 8-second (s) rpm; the speed was gradually increased from 4 rpm to a maximum of 40 rpm until the rat dropped from the shaft, and the machine displayed the rat's running time. The test was repeated five times, and the three highest scores were selected and averaged.

#### 2.4.3. Passive Avoidance Test

A passive avoidance test was performed using the GEMINI Avoidance System (San Diego Instruments, San Diego, CA, USA), and the test consists of dark (left) and light rooms (right) of the same size (25 × 20 × 17 cm) connected by a gate (9 × 7 cm) in the middle. The floor of each room comprises 14 stainless steel rods (6 mm in diameter) with 1.8 cm spacing, and the rods are connected to a shock scrambler.

- Habituation test (habituation): the habituation test was performed 1 day before right MCA occlusion. The rat was placed in the light room. After 5 s, the light of the room was turned on and the gate to the room was opened simultaneously. Once the rat entered the dark room, the gate was closed immediately. The time taken to enter the dark room was recorded. Thirty seconds after the gate was closed, the rat was taken out and returned to its cage. If the rat took more than 100 s to enter the dark room, it was eliminated from analysis. The whole process was repeated after 30 min, and the response with the highest number of seconds was selected.
- Training session: the training session was executed 1 h before right MCA occlusion. The rat was placed in the light room. After 5 s, the light of the room was turned on and the gate to the room was opened simultaneously. Once the rat entered the dark room, the gate was immediately closed, and the rat received an intermittent electric shock (50 Hz, 3 s, 0.5 mA). After 30 s, the rat was placed back into its cage. The time taken to enter the dark room was recorded. If the rat stayed in the light room for more than 120 s, the training was terminated and the reading was considered to be 120 s. The training process was repeated after 2 min, and the response with the highest number of seconds was selected.
- Memory test (retention trial): this test was performed on days 3 and 15 after reperfusion. The rat was placed in the light room. After 5 s, the light of the room was turned on and the gate to the room was opened simultaneously. If the rat did not enter the dark room in >300 s, the test was terminated and recorded as 300 s. The whole process was performed three times, with a 2-minute intertest interval, and the test with the highest number of seconds was selected.

### 2.5. The Measurement of Cerebral Infarction Volume

On day 15 after reperfusion, the rats' brains were removed under 4% isoflurane anesthesia description as in our previous study [21]. Then the rats' brains were put into a plastic model of the rat brain and cut into 6 pieces of 2 mm thickness from the frontal pole. The slices were stained with TTC (Merk, Germany) for 15 minutes. The infarction region showed white color, whereas noninfarction region was purple-red. The cerebral infarction size was decided by using a microscopic image-analysis system (Image-Pro Lito Version 3.0, Media Cybernetics, USA). Each slice ratio of the infarction volume to the total brain was calculated, and data are presented as percentage (%).

### 2.6. Western Blot Analysis

The rats' brains were divided into the cerebral cortex and hippocampus in the left and right hemispheres.

First, lysis buffer (50 mM Tris-HCl, 0.5% Triton X-100, 1X protease inhibitor) at 2.5x the tissue weight was added to the brain tissue, and the mixture was sonicated and centrifuged at 15,000 rpm and 4°C for 10 min. The supernatant was collected and stored at −80°C.

Sodium dodecyl sulphate (SDS) sample buffer (62.5 mM Tris-HCl at pH6.8, 2% SDS, 10% glycerol, 50 mM dithiothreitol (DTT), and 0.1% bromophenol blue) was used to obtain tissue cell extract. Next, 20 *μ*g of protein was taken for 10% SDS-polyacrylamide (PAGE) analysis and transferred to nitrocellulose (NC) paper. After blocking the NC paper with 5% skimmed milk for 1 h, primary anti-GABA-A receptor antibodies, brain-derived neurotrophic factor (BDNF), and postsynaptic density protein 95 (PSD-95) were added. The sample was allowed to incubate overnight at 4°C. After adding a secondary antibody, the mixture was incubated at room temperature for 1 h. Finally, the microfluidic electrochemiluminescence (ECL) color rendering system (Amersham) was added for color development, and the film was exposed and developed under cold light. The present study uses AlphaEaseFC software to calculate the integrated density valve (IDV) and then divides it by Actin to obtain the value of Western blot data.

### 2.7. Statistical Analysis

The data are represented as median (quartile 1, quartile3) or mean ± standard deviation. The Kruskal-Wallis nonparametric test, with Dunn's post hoc test, was used for neurological deficit score, Rotarod test, and passive avoidance test. The ANOVA test with Games-Howell post hoc test was used for GABA-A, and Tukey's HSD post hoc test was used for BDNF and PSD-95. *p* < 0.05 was considered statistically significant.

## 3. Results

### 3.1. Effect of 2 Hz EA at Contralesional BL8 and BL7 Acupoints on the Neurological Deficit Score in Ischemia-Reperfusion Injury Rats

On day 3 after reperfusion, the neurological deficit scores were similar in the sham and EA groups (7.00 (7.00, 8.00) versus 7.00 (7.00, 8.00), *p* > 0.05; Table 2), but both were greater than that in the normal group (0.00 (0.00, 0.00), both *p* < 0.001; Table 2). The difference in the neurological deficit score between days 3 and 15 after reperfusion was 0.00 (−1.00, 0.00) in the sham group, which was less than −4.00 (−4.00, −3.00) in the EA group (*p* < 0.001; Table 2), but was similar to 0.00 (0.00, 0.00) in the normal group (*p* > 0.05). The score of the EA group was larger than that of the normal group (*p* < 0.001; Table 2).

### 3.2. Effect of 2 Hz EA at Contralesional BL8 and BL7 Acupoints on the Rotarod Test in Ischemia-Reperfusion Injury Rats

On day 3 after reperfusion, the Rotarod test results were similar in the sham and EA groups (34.22 (28.31, 42.77) s versus 37.52 (32.72, 42.73) s, *p* > 0.05; Table 2), but scores in both were less than that in the normal group (85.92 (81.54, 133.56) s, both *p* < 0.001; Table 2). On day 15 after reperfusion, the difference in Rotarod test time between day 15 and day 3 was 3.53 (−0.39, 7.48) s in the sham group, which was less than that in the EA group (44.80 (41.69, 54.13) s, *p* < 0.001; Table 2) but similar to that in the normal group (2.57 (−7.93, 7.78) s, *p* > 0.05; Table 2). The EA group had a higher Rotarod test time compared to the normal group (*p* < 0.001; Table 2).

### 3.3. Effect of 2 Hz EA at Contralesional BL8 and BL7 Acupoints on Passive Avoidance Test in Ischemia-Reperfusion Injury Rats

On day 3 after reperfusion, the retention times of the passive avoidance test were similar in the normal, sham, and EA groups (226.40 (33.43, 300.00) s, 300.00 (243.60, 300.00) s, and 300.00 (138.90, 300.00) s, respectively, all *p* > 0.05; Table 2). The difference in the retention time of the passive avoidance test between days 15 and 3 was −168.75 (−275.85, −8.25) s in the sham group, −28.25 (−135.43, 0) s in the EA group, and −8.05 (−53.55, 10.05) s in the normal group, and the differences were not significant (all *p* > 0.05; Table 2).

### 3.4. Effect of 2 Hz EA at Contralesional BL8 and BL7 Points on Cerebral Infarction Volume in Ischemia-Reperfusion Injury Rats

**Figure 1:**
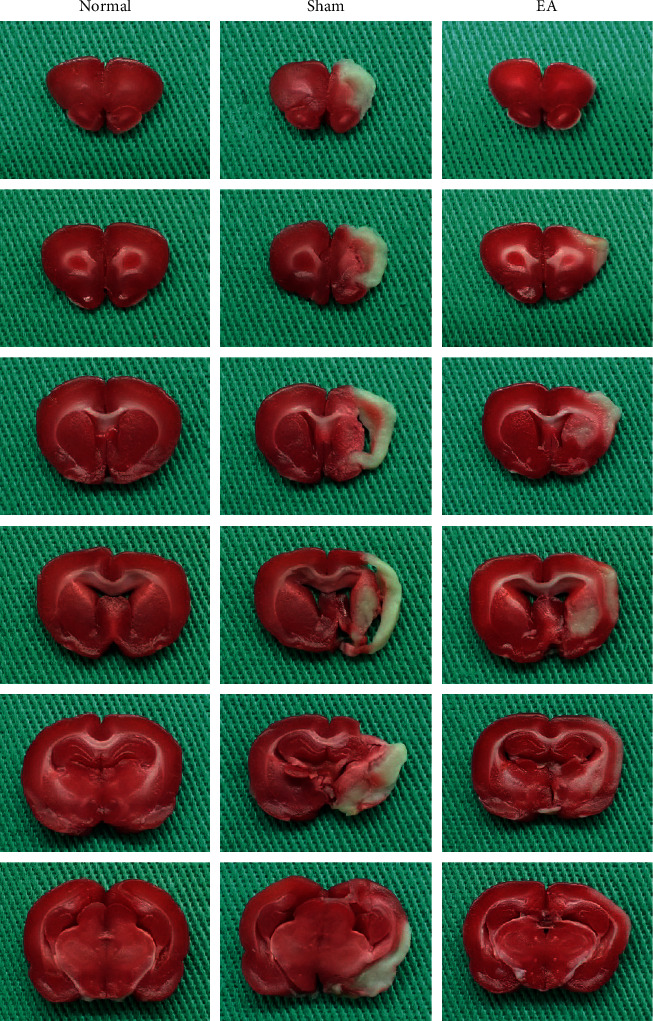
Effect of 2 Hz EA at contralesional BL8 and BL7 points on infarction volume in ischemia-reperfusion injury rats. Brain tissue used 2,3,5-triphenyltetrazolium chloride (TTC) stain. The ratio of cerebral infarction volume in the electroacupuncture group (EA) was less than that in the sham group (Sham). Normal: normal group; purple-red color: normal brain tissue; white color: cerebral infraction region.

On day 15 after reperfusion, the ratio of infarction volume in the normal group was 0.00 ± 0.00, which was less than 0.19 ± 0.04 in the sham group (*p* < 0.001; Table 2 and Figure 1) and 0.07 ± 0.04 in the EA group (*p* < 0.01; Table 2 and Figure 1). The ratio in the sham group was greater than that in the EA group (*p* < 0.001; Table 2 and Figure 1).

### 3.5. Effect of 2 Hz EA at Contralesional BL8 and BL7 Points on GABA-A, BDNF, and PSD-95 in the Cerebral Cortex in Rats with Ischemic Stroke

Western blot analysis of brain tissue was performed on day 15 after reperfusion.

**Figure 2:**
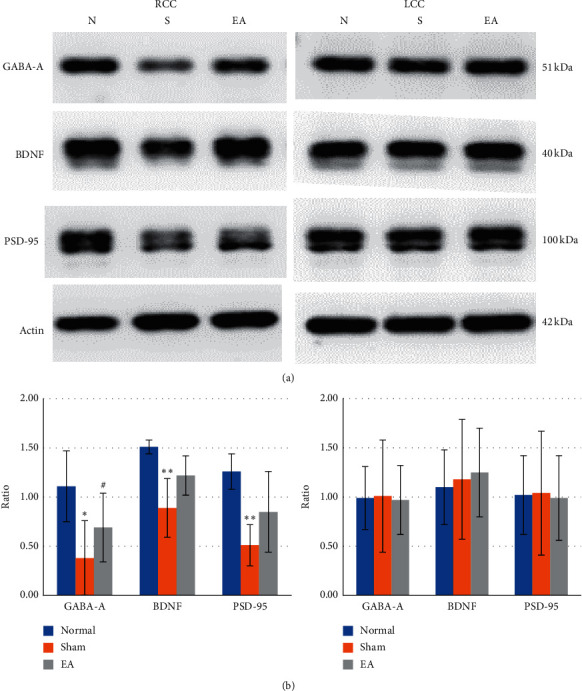
Effect of 2 Hz EA at contralesional BL8 and BL7 points on GABA-A, BDNF, and PSD-95 in the cerebral cortex in ischemia-reperfusion injury rats. Western blot analysis at day 15 after reperfusion indicated that the GABA-A, BDNF, and PSD-95 levels were reduced in the cerebral cortex of the lesioned hemisphere (right hemisphere), and the reduction of GABA-A was restored by 2 Hz EA applied on the BL8 and BL7 points of the contralesional scalp. RCC: cerebral cortex of lesioned hemisphere; LCC: cerebral cortex of nonlesioned hemisphere (left hemisphere); *N* or Normal: normal group; *S* or Sham: sham group; EA: electroacupuncture group; ^*∗*^*p* < 0.05 and ^*∗∗*^*p* < 0.01 compared to normal; ^#^*p* < 0.05 compared to sham.

In the cerebral cortex of the lesioned hemisphere (right hemisphere), the GABA-A/actin ratio in the normal group was greater than that in the sham group (1.11 ± 0.36 versus 0.38 ± 0.07, *p* < 0.05; Figures 2(a) and 2(b), left) and similar to that in the EA group (0.69 ± 0.18, *p* > 0.05; Figures 2(a) and 2(b), left). The ratio in the EA group was larger than that in the sham group (*p* < 0.05; Figures 2(a) and 2(b), left).

The BDNF/actin ratio in the normal group was greater than that in the sham group (1.51 ± 0.38 versus 0.89 ± 0.30, *p* < 0.01; Figures 2(a) and 2(b), left) and similar to that in the EA group (1.22 ± 0.21, *p* > 0.05; Figures 2(a) and 2(b), left). The ratios in the EA and sham groups were not significantly different (*p* > 0.05; Figures 2(a) and 2(b), left).

The PSD-95/actin ratio in the normal group was greater than that in the sham group (1.26 ± 0.35 versus 0.51 ± 0.20, *p* < 0.01; Figures 2(a) and 2(b), left) and similar to that in the EA group (0.85 ± 0.41, *p* > 0.05; Figures 2(a) and 2(b), left). The ratios in the EA and sham groups were not significantly different (*p* > 0.05; Figures 2(a) and 2(b), left).

In the cerebral cortex of the nonlesioned hemisphere (left hemisphere), the GABA-A/actin ratio in the normal, sham, and EA groups was 0.99 ± 0.32, 0.01 ± 0.38, and 0.97 ± 0.40, respectively, and the differences were not significant (all *p* > 0.05; Figures 2(a) and 2(b), right).

The BDNF/actin ratio in the normal, sham, and EA groups was 1.18 ± 0.57, 1.25 ± 0.63, and 1.17 ± 0.51, respectively, and the differences were not significant (all *p* > 0.05; Figures 2(a) and 2(b), right).

The PSD-95/actin ratio in the normal, sham, and EA groups was 1.02 ± 0.35, 1.04 ± 0.45, and 0.99 ± 0.39, respectively, and the differences were not significant (all *p* > 0.05; Figures 2(a) and 2(b), right).

### 3.6. Effect of 2 Hz EA at Contralesional BL8 and BL7 Acupoints on GABA-A, BDNF, and PSD-95 in the Hippocampus in Rats with Ischemic Stroke

**Figure 3:**
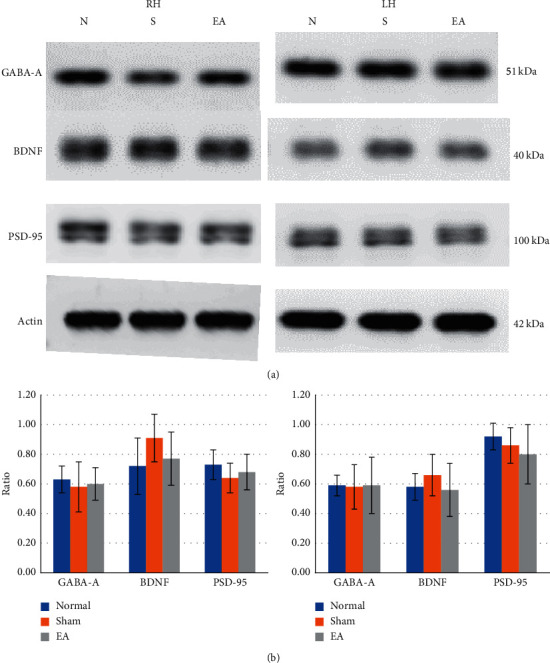
Effect of 2 Hz EA at contralesional BL8 and BL7 points on GABA-A, BDNF, and PSD-95 in the thalamus in ischemia-reperfusion injury rats. Western blot analysis at day 15 after reperfusion showed no prominent differences in GABA-A, BDNF, and PSD-95 levels among the normal, sham, and EA groups in the hippocampus of the lesioned hemisphere and nonlesioned hemisphere. RH: hippocampus of lesioned hemisphere; LH: hippocampus of nonlesioned hemisphere; *N* or Normal: normal group; *S* or Sham: sham group; EA: electroacupuncture group.

In the lesioned hippocampus (right hemisphere), the GABA-A/actin ratio in the normal, sham, and EA groups was 0.63 ± 0.09, 0.58 ± 0.19, and 0.60 ± 0.10, respectively, and the differences were not significant (all *p* > 0.05; Figures 3(a) and 3(b), left).

The BDNF/actin ratio in the normal, sham, and EA groups was 0.72 ± 0.17, 0.91 ± 0.16, and 0.77 ± 0.10, respectively, and the differences were not significant (all *p* > 0.05; Figures 3(a) and 3(b), left).

The PSD-95/actin ratio in the normal, sham, and EA groups was 0.73 ± 0.11, 0.64 ± 0.18, and 0.68 ± 0.12, respectively, and the differences were not significant (all *p* > 0.05; Figures 3(a) and 3(b), left).

In the hippocampus of the nonlesioned hemisphere (left hippocampus), the GABA-A/actin ratio in the normal, sham, and EA groups was 0.59 ± 0.07, 0.58 ± 0.15, and 0.59 ± 0.09, respectively, and the differences were not significant (all *p* > 0.05; Figures 3(a) and 3(b), right).

The BDNF/actin ratio in the normal, sham, and EA groups was 0.58 ± 0.15, 0.66 ± 0.14, and 0.56 ± 0.12, respectively, and the differences were not significant (all *p* > 0.05; Figures 3(a) and 3(b), right).

The PSD-95/actin ratio in the normal, sham, and EA groups was 0.92 ± 0.19, 0.86 ± 0.18, and 0.80 ± 0.20, respectively, and the differences were not significant (all *p* > 0.05; Figures 3(a) and 3(b), right).

## 4. Discussion

The results of the present study revealed that the difference in both the neurological deficit score and Rotarod test time between days 3 and 15 after reperfusion was greater in the EA group than in the sham group, whereas the difference in the retention time of the passive avoidance test was not significantly different. In addition, the infarction volume also was lesser in the EA group than in the sham group. This suggests that 2 Hz EA applied to BL8 and BL7 acupoints of the contralesional scalp can improve neurological deficit but not learning memory in ischemia-reperfusion injury rats. Moreover, on day 15 after reperfusion, the GABA-A levels in the cerebral cortex of the lesioned hemisphere (right hemisphere) decreased, but 2 Hz EA on the BL8 and BL7 points of the contralesional scalp could restore these decreases, indicating that GABA-A plays a critical role in poststroke interhemispheric imbalance. If the inhibition from the lesioned to nonlesioned hemisphere is reduced, this results in excitation of the contralesional hemisphere, leading to a greater inhibition of the lesioned hemisphere [10]. Our results were similar to several studies showing that training the nonparetic (intact) forelimb affects the functional recovery of the impaired forelimb in early stage of endothelial-1 induced focal ischemic rat model, but this phenomenon does occur in rat with transection of corpus callosum, suggesting that the training of intact forelimbs may affect the neural activity peri-infarct area through corpus callosum [22, 23]. The GABA-A agonist midazolam can attenuate long-latency interhemispheric inhibition [24]. Taken together, the findings suggest that 2 Hz EA at contralesional BL8 and BL7 acupoints of the scalp increased GABA-A levels, promoting the recovery of poststroke interhemispheric imbalance.

Furthermore, on day 15 after reperfusion, BDNF and PSD-95 levels in the cerebral cortex of the lesioned hemisphere were reduced and did not increase with 2 Hz EA on BL8 and BL7 points of the contralesional scalp. BDNF is widely distributed in the central nervous system and intestines and plays a vital role in the survival and growth of neuronal cells. It acts as a mediator of neurotransmitters, and the plasticity of neuronal cells is essential for learning and memory [25]. Lower serum BDNF levels in the early stage after stroke are a factor for poor recovery after stroke [26]. BDNF also plays a key role in rehabilitation to promote recovery from stroke [27]. PSD-95 regulates NMDA in the nervous system. Intravenous injection of PSD-95 inhibitors (Tat-NR2B9c _(SDV)_ or Tat-NR2B9c _(TDV)_) 3 h after stroke can reduce infarct size and improve neurological function in a stroke with a hyperthermic rat model [28]. Drugs such as Zl006 (5-(3,5-dichloro-2-hydroxybenzylamino)-2-hydroxybenzoic acid) which can inhibit the formation of the neuronal nitric oxide synthase-PSD-95 complex can play a neuroprotective role in stroke treatment [29, 30]. However, the relation between BDNF and PSD-95 and 2 Hz EA at BL8 and BL7 acupoints of the contralesional scalps needs further study.

Our results also demonstrated that 2 Hz EA at BL8 and BL7 points of the contralesional scalp could not change the levels of GABA-A, BDNF, and PSD-95 in the cerebral cortex of the contralesional hemisphere and in the hippocampus of the lesioned and contralesional hemisphere. One study reported that repetitive transcranial magnetic stimulation over the contralesional motor cortex may reduce transcallosal inhibition and thus improve the affected hand in patients with stroke [31]. In addition, stroke lesions can indirectly break off interhemispheric inhibition, contributing to poststroke recovery of motor function [32]. The corpus callosum plays a role in information integration across both cerebral hemispheres and serves as inhibition or excitation, even simultaneously depending on the task [33]. We speculate that 2 Hz EA at BL8 and BL7 acupoints of contralesional scalp can reduce inhibition from the cerebral cortex of the contralesional hemisphere via the corpus callosum, not via the anterior or hippocampal commissure to the cerebral cortex of the lesioned hemisphere. This is consistent with our results, where GABA-A levels increased in the cerebral cortex but not in the hippocampus, as the corpus callosum mainly interconnects a large part of the cerebral cortex [34], but this hypothesis requires further verification.

There are some limitations in the present study. First, the present study only used 2 Hz EA, more frequencies of EA such as 15 Hz will be studied; second, the EA was applied to contralesional hemisphere (BL8 and BL7) only; EA applied to bilateral BL8 and BL7 simultaneously or EA applied to scalp plus body will be investigated; third, neurotransmitter was studied in the brain tissue only; the changes of neurotransmitters in the cerebrospinal fluid will be studied in the future.

In conclusion, our results revealed that 2 Hz EA applied at the BL8 and BL7 acupoints of the contralesional scalp improved the neurological deficit score, reduced infarction volume, increased the Rotarod test time, and increased the GABA-A levels in the cerebral cortex of the lesioned hemisphere of the ischemia-reperfusion injury rats, suggesting that 2 Hz EA improves motor function and that GABA-A plays at least a partial role.

## Figures and Tables

**Table 1:** The evaluation of neurological deficit score.

Test	Scores
*Motor test*	0–6
(1) Raising the rat by the tail	0–3
(2) Placing the rat on the floor	0–3

*Sensory test*	0–2
(1) Placing test	0–1
(2) Proprioceptive test	0–1
*Beam balance test*	0–6

*Reflex test*	0–4
(1) Pinna reflex	0–1
(2) Corneal reflex	0–1
(3) Startle reflex	0–1
(4) Seizure, myoclonus, myoclonia	0–1
Total score	18

**Table 2:** The effect of electroacupuncture on neurological status and infarction volume in ischemia-reperfusion injury rats.

	Group		
	Normal (*N* = 12)	Sham (*N* = 12)	EA (*N* = 12)
Neurological deficit score			
D3	0.00 (0.00, 0.00)	7.00 (7.00, 8.00)^*∗∗∗*^	7.00 (7.00, 8.00)^*∗∗∗*^
D15	0.00 (0.00, 0.00)	7.00 (7.00, 7.00)^*∗∗∗*^	4.00 (3.00, 4.00)^*∗*#^
Day 14–day 3	0.00 (0.00, 0.00)	0.00 (−1.00, 0.00)	−4.00 (−4.00, −3.00)^*∗∗∗*###^

Rotarod test			
D3	85.92 (81.54, 133.56)	34.22 (28.31, 42.77)^*∗∗∗*^	37.52 (32.72, 42.73)^*∗∗∗*^
D15	91.78 (88.78, 105.34)	38.35 (28.97, 42.43)^*∗∗∗*^	84.10 (76.83, 92.43)^##^
D15–D3	2.57 (−7.93, 7.78)	3.53 (−0.39, 7.48)	44.80 (41.69, 54.13)^*∗∗∗*###^

Passive avoidance test			
D3	226.40 (33.43, 300.00)	300.00 (243.60, 300.00)	300.00 (138.90, 300.00)
D15	167.00 (16.40, 300.00)	49.00 (16.80, 276.93)	271.75 (114.68, 300.00)
D15–D3	−8.05 (−53.55, 10.05)	−168.75 (−275.85, −8.25)	−28.25 (−135.43, 0.00)

Infarction volume			
Ratio (*N* = 6)	0.00 ± 0.00	0.19 ± 0.04^*∗∗∗*^	0.07 ± 0.04^*∗∗*###^

Data are represented as median (Q1, Q3) or mean ± standard deviation. Normal: normal group; Sham: sham group; EA: electroacupuncture group; D3: 3^rd^ day after reperfusion; D15 : 15^th^ day after reperfusion; D15–D3: the difference between the 15^th^ day and 3^rd^ day; ^*∗*^*p* < 0.05, ^*∗∗*^*p* < 0.01, and ^*∗∗∗*^*p* < 0.001 compared to normal; ^#^*p* < 0.05, ^##^*p* < 0.01, and ^###^*p* < 0.001 compared to sham.

## Data Availability

The data used in this study are available to other researchers upon request.
